# Ureteroinguinal hernia with obstructive urolithiasis

**DOI:** 10.1590/S1677-5538.IBJU.2019.0415

**Published:** 2020-07-31

**Authors:** JuliAnne R. Rathbun, Nanda Thimmappa, Stephen H. Weinstein, Katie S. Murray

**Affiliations:** 1 University of Missouri School of Medicine Division of Urology Columbia Missouri USA Division of Urology, University of Missouri School of Medicine, Columbia, Missouri, USA; 2 University of Missouri School of Medicine Department of Surgery Columbia Missouri USA Department of Surgery University of Missouri School of Medicine, Columbia, Missouri, USA; 3 University of Missouri School of Medicine Department of Radiology Columbia Missouri USA Department of Radiology, University of Missouri School of Medicine, Columbia, Missouri, USA

## CASE REPORT

A 64-year-old male was referred for elevated PSA of 13.7ng/mL. He underwent transrectal ultrasound-guided prostate biopsy and was found to have Gleason 4+4 prostate cancer. Staging computed tomography (CT) revealed mild left renal atrophy and left hydroureteronephrosis. The dilated ureter extended down through the left inguinal canal and into the left hemiscrotum, where a 1cm stone was noted within a ureteroinguinal hernia (Figure I). The right ureter was also contained within a right ureteroinguinal hernia, but was not dilated. Bone scan showed retained contrast in the left distal ureter within the hernia (Figure II) . The patient endorsed mild back pain that he attributed to lifting and physical activity. He had a history of hypertension, and his creatinine had elevated to 1.7mg/dL over the last two years. He was evaluated by general surgery, and his bilateral inguinal hernias were noted to be non-palpable.

**Figure 1:**
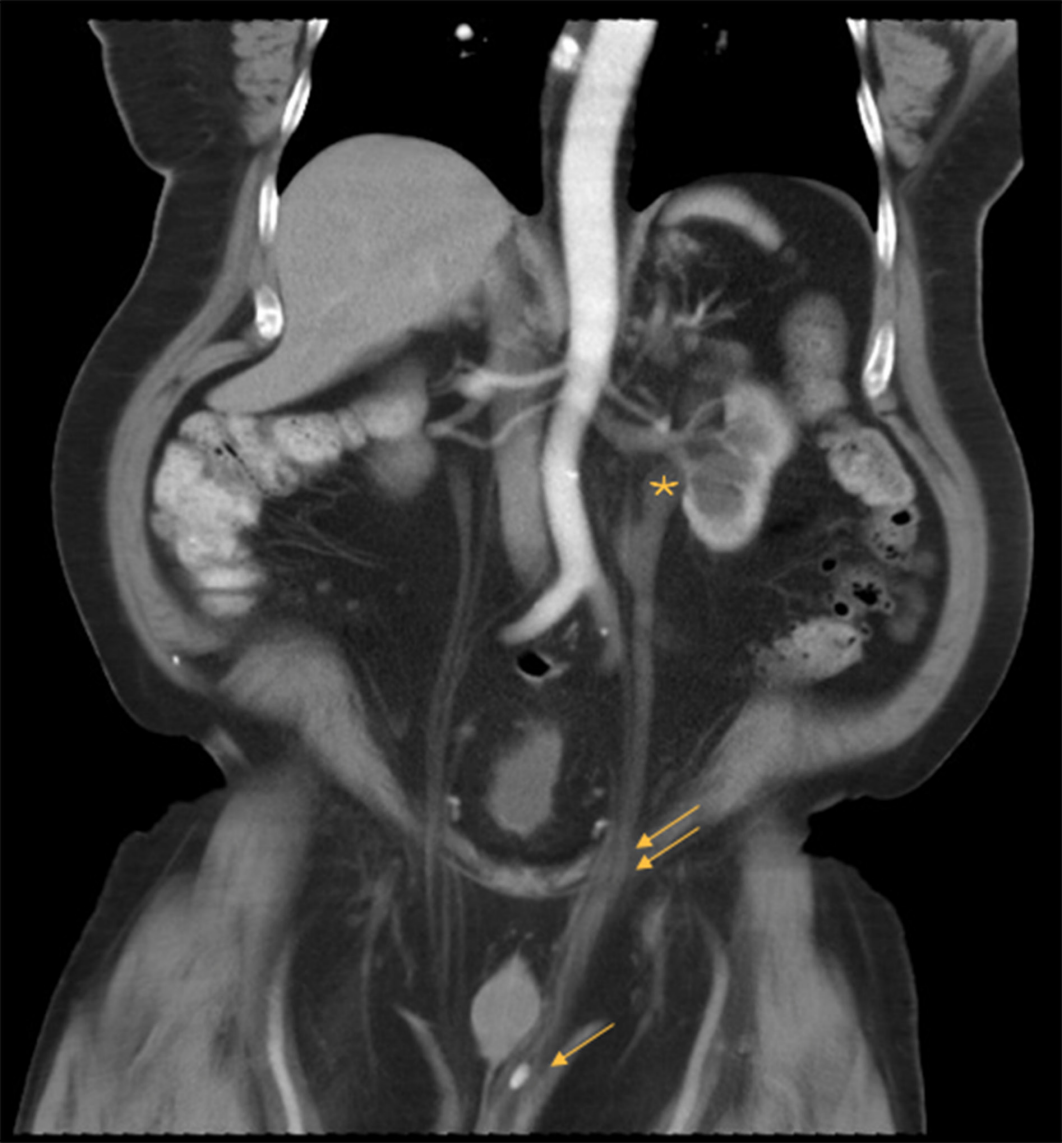
Coronal contrast-enhanced CT in nephrographic phase demonstrating left hydroureteronephrosis (*). Dilated left ureter noted to pass through inguinal canal (double arrows) and into the left hemiscrotum containing a stone (single arrow).

**Figure 2:**
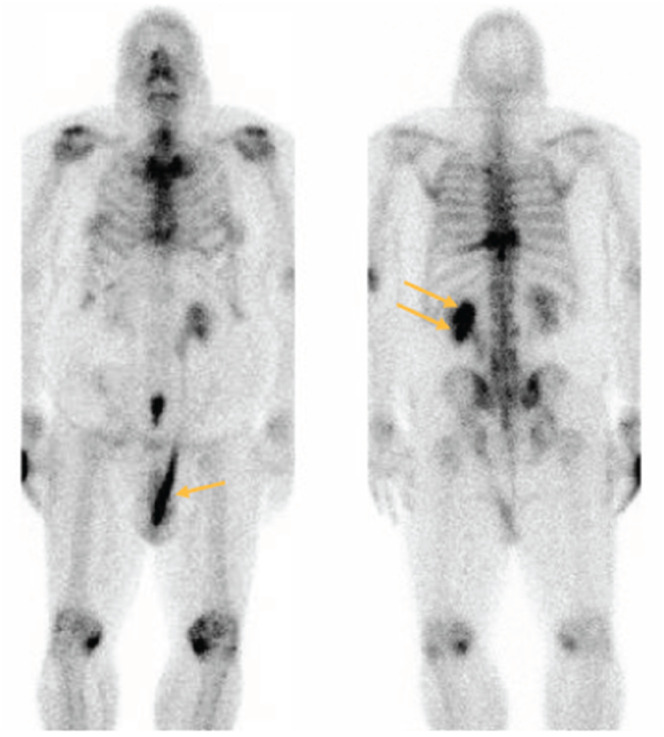
Coronal bone scan showing retained contrast within left scrotal ureter (arrow) on the anterior view and slow drainage from the left kidney (double arrow) on the posterior view.

Inguinal hernias can be direct or indirect and have the lifetime risk of development of 27-43% in men and 3-6% in women (1–3). Risk factors for inguinal hernia development include increased age, low body mass index (BMI) and genetic mutations altering connective tissue (1). Indirect hernia risk factors are patent processus vaginalis and increased cumulative occupational mechanical exposure (1). A unique subdivision of indirect inguinal hernias is ureteroinguinal. Of the two types of ureteroinguinal hernias, the most common are paraperitoneal (80%), which are associated with a peritoneal evagination (4, 5). Extraperitoneal ureteroinguinal hernias involve the ureter alone or with retroperitoneal fat (4–6). In the literature, around 140 cases have been described, and very few of these with obstructive uropathy (6, 7). Management involves herniorrhaphy with a team-based approach between general surgery and urology (8). Risk of recurrence after standard repair increases with elevated intraabdominal pressures, which can be secondary to high BMI (1).

The patient underwent bilateral laparoscopic inguinal hernia repair with subsequent left ureteroscopy for his stone. His ureter was noted to be extremely elongated and tortuous after hernia repair. His creatinine peaked at 2.3mg/dL at time of hernia repair, and then it improved to 1.6mg/dL by the time of ureteroscopy. He has recovered well from both surgeries and is planning to undergo fluciclovine F-18 scan for further staging of his prostate cancer. His hydroureteronephrosis was persistent on his immediately post-operative CT scan.
